# Mental Health In Elite Athletes: Increased Awareness Requires An Early Intervention Framework to Respond to Athlete Needs

**DOI:** 10.1186/s40798-019-0220-1

**Published:** 2019-11-28

**Authors:** Rosemary Purcell, Kate Gwyther, Simon M. Rice

**Affiliations:** 1 Orygen, The National Centre of Excellence in Youth Mental Health, 35 Poplar Road, Parkville, Melbourne, Victoria 3052 Australia; 2 Centre for Youth Mental Health, University of Melbourne, Melbourne, Victoria Australia

## Abstract

The current ‘state of play’ in supporting elite athlete mental health and wellbeing has centred mostly on building mental health literacy or awareness of the signs of mental ill-health amongst athletes. Such awareness is necessary, but not sufficient to address the varied mental health needs of elite athletes. We call for a new model of intervention and outline the backbone of a comprehensive mental health framework to promote athlete mental health and wellbeing, and respond to the needs of athletes who are at-risk of developing, or already experiencing mental health symptoms or disorders. Early detection of, and intervention for, mental health symptoms is essential in the elite sporting context. Such approaches help build cultures that acknowledge that an athlete’s mental health needs are as important as their physical health needs, and that both are likely to contribute to optimising the athlete’s overall wellbeing in conjunction with performance excellence. The proposed framework aims at (i) helping athletes develop a range of self-management skills that they can utilise to manage psychological distress, (ii) equipping key stakeholders in the elite sporting environment (such as coaches, sports medicine and high-performance support staff) to better recognise and respond to concerns regarding an athlete’s mental health and (iii) highlighting the need for specialist multi-disciplinary teams or skilled mental health professionals to manage athletes with severe or complex mental disorders. Combined, these components ensure that elite athletes receive the intervention and support that they need at the right time, in the right place, with the right person.

**Key Points**
- Currently, there is no comprehensive framework or model of care to support and respond to the mental health needs of elite athletes.
- We propose a framework that recognises the impact of general and athlete-specific risk factors, and engages key individuals that may identify and promote athlete mental health.
- The framework is adaptable and responsive to varied career stages and mental health states.

There has been a rapid increase in research examining the mental health of elite athletes culminating with the International Olympic Committee’s (IOC’s) recent Expert Consensus Statement on mental health in elite athletes [1]. This statement provides a comprehensive analysis of, and recommendations for, the treatment of both high prevalence (e.g. anxiety and mood symptoms) and more complex mental health disorders (e.g. eating and bipolar disorders) in the elite sporting context. This is a timely resource which will help guide and ultimately improve the clinical management of athletes by sports medicine, mental health, and allied health professionals. The primary focus of the consensus statement, along with much of the extant literature, is on managing the individual athlete affected by mental ill-health. There has been little scholarly and service-level attention to more comprehensive frameworks that (a) recognise the role of the broader elite sports ecology as both a contributor to athlete mental health difficulties and a facilitator of their remediation, and (b) approaches that emphasise the *prevention* of mental health symptoms, along with early detection and intervention to restore athlete wellbeing (and ideally optimise performance).

## Risk Factors for Mental Ill-health in Elite Athletes

Meta-analytic reviews indicate that elite athletes experience broadly comparable rates of mental ill-health relative to the general population in relation to anxiety, depression, post-traumatic stress and sleep disorders [2, 3]. This should not be unexpected given the considerable overlap in the years of active elite competition and the primary ages of onset for most mental disorders [4–6].

Increasing evidence points to a range of both athlete-specific and general risk factors associated with mental ill-health in elite athletes. Athlete-specific risk indicators include sports-related injury and concussion [3, 7–9], performance failure [10], overtraining (and overtraining syndrome) [11] and sport type (e.g. individual sports conferring a higher risk that team sports) [12]. General risk indicators include major negative life events [13, 14], low social support [15, 16] and impaired sleep [17, 18]. These risk factors may impact the severity and onset of particular mental health symptoms, but can also guide appropriate response strategies.

The salience of particular risk factors may vary across career phases. For example, in junior development years, supportive relationships with parents and coaches are imperative to athlete wellbeing [19, 20]. During the high performance and elite phase, in addition to the coaching relationship, environmental and training demands become more relevant to mental health and wellbeing [21], including extended travel away from home and exposure to unfamiliar (training) environments [22]. Environmental conditions and travel may be especially salient for the mental health of para-athletes, who often encounter disruptive logistical issues associated with travel, such as a lack of adaptive sport facilities and sleeping conditions [23]. Prominent risk factors during the transition out of sport include involuntary or unplanned retirement and lack of a non-athletic identity, both of which are associated with a range of psychological challenges [24]. For para-athletes, involuntary retirement due to declassification (i.e. no longer meeting the required criteria to be classified as a para-athlete) is a unique burden [25].

## Optimising the Mental Health and Wellbeing of Elite Athletes: Barriers and Facilitators

A comprehensive framework for mental health in elite athletes needs to consider the range of relevant risk factors across key career phases, as well as factors that inhibit or facilitate the ability to effectively respond to athletes’ needs. Key barriers include more negative attitudes towards help-seeking amongst athletes than the general population [26], as well as greater stigma and poorer mental health literacy. Fear of the consequences of seeking help (e.g. loss of selection) and lack of time are also influential [26–28]. Facilitative factors include support and acknowledgment from coaches [27] who can help to create a non-stigmatised environment where help-seeking can be normalised [28]. Approaches that seek to optimise athletic performance while simultaneously providing intervention for mental health symptoms may also facilitate engagement [29, 30]. Brief anti-stigma interventions and mental health literacy programs that seek to increase knowledge of mental health symptoms have been shown to improve help-seeking intentions in elite athletes [31–33], although the impact of such programs on help-seeking *behaviours* is not known.

## Are there Existing Frameworks or Models of Care for Mental Health in Elite Sport?

To date there are no published frameworks regarding how best to support the mental health needs of elite athletes. In addition to the IOC Consensus Statement, recent position statements have emphasised the need to build awareness of mental health problems and increase help-seeking behaviours [34–36]. These initiatives are unquestionably warranted; however, improving awareness and help-seeking behaviours are at best pointless, and at worst unsafe, if systems of care to respond to athlete’s need are not available. A whole of system approach needs to be developed simultaneously.

Beyond the peer-reviewed literature, useful guidelines exist within selected sporting associations regarding supporting athlete wellbeing [37–39]. These resources highlight a number of critical factors in managing athlete mental health in the sporting context including (i) the sports’ responsibility for managing the athlete’s care and support (e.g. duty of care issues); (ii) the need for regular screening or monitoring of athletes to detect changes in mental state or behaviour; (iii) privacy and confidentiality regarding mental health as key ethical issues and challenges; (iv) athlete preferences for help-seeking (how and from whom); (v) the need to refer out to or engage external mental health professionals where expertise does not exist within the sporting environment; and (vi) the value of trained peer workers (former athletes/players) to provide support and guidance to athletes and to coordinate activities related to professional development needs (such as public speaking or financial planning) and individual goal-setting (e.g. around educational or post-sport vocational interests). However, no single framework incorporates all of these factors nor is there a framework that focuses on the *spectrum* of athlete/player mental health needs, from symptom prevention to specialist mental health care. There has been some progress in developing mental health guidelines in collegiate-level athletes [40–42], which highlight the need to provide specific and targeted support, while noting that few comprehensive or targeted models of care for mental health have been developed for this population.

## Developing a Comprehensive Mental Health Framework to Support Elite Athletes

**Fig 1.**
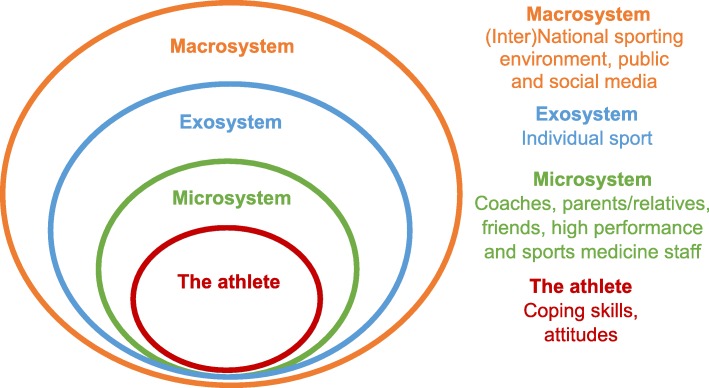
An ecological systems model for elite athlete mental health

Many of the general and athlete-specific risk factors for mental ill-health are potentially modifiable (e.g. coping strategies, coaching style, training demands) and require intervention at the individual athlete, the sporting or environmental and/or organisational levels. A comprehensive framework for athlete mental health that is conceptualised within the broader ‘ecology’ of elite sporting environments will be best able to respond to the range of risk indicators in this context (see Fig. 1). Ecological systems help to explain the relationship between the aspects or experiences of an individual (termed ‘ontogenetic’ factors, such as coping or substance use) and the broader social and cultural contexts in which they exist [43]. In the case of elite athletes, this includes the ‘microsystem’ of coach(es), teammates (where appropriate) and family/loved ones. The wider sporting environment (e.g. the athlete’s sport, its rules and governing body) forms the exosystem, while the role of national and international sporting bodies and the media and broader society form the macrosystem.

Any mental health framework that ignores wider ecological factors runs the risk of focusing exclusively on, and potentially pathologising the individual athlete, when other factors may be more influential in contributing to, or perpetuating poor mental health. Such factors may include maladaptive relationships with coaches or parents, social media abuse and/or financial pressures.

**Fig 2.**
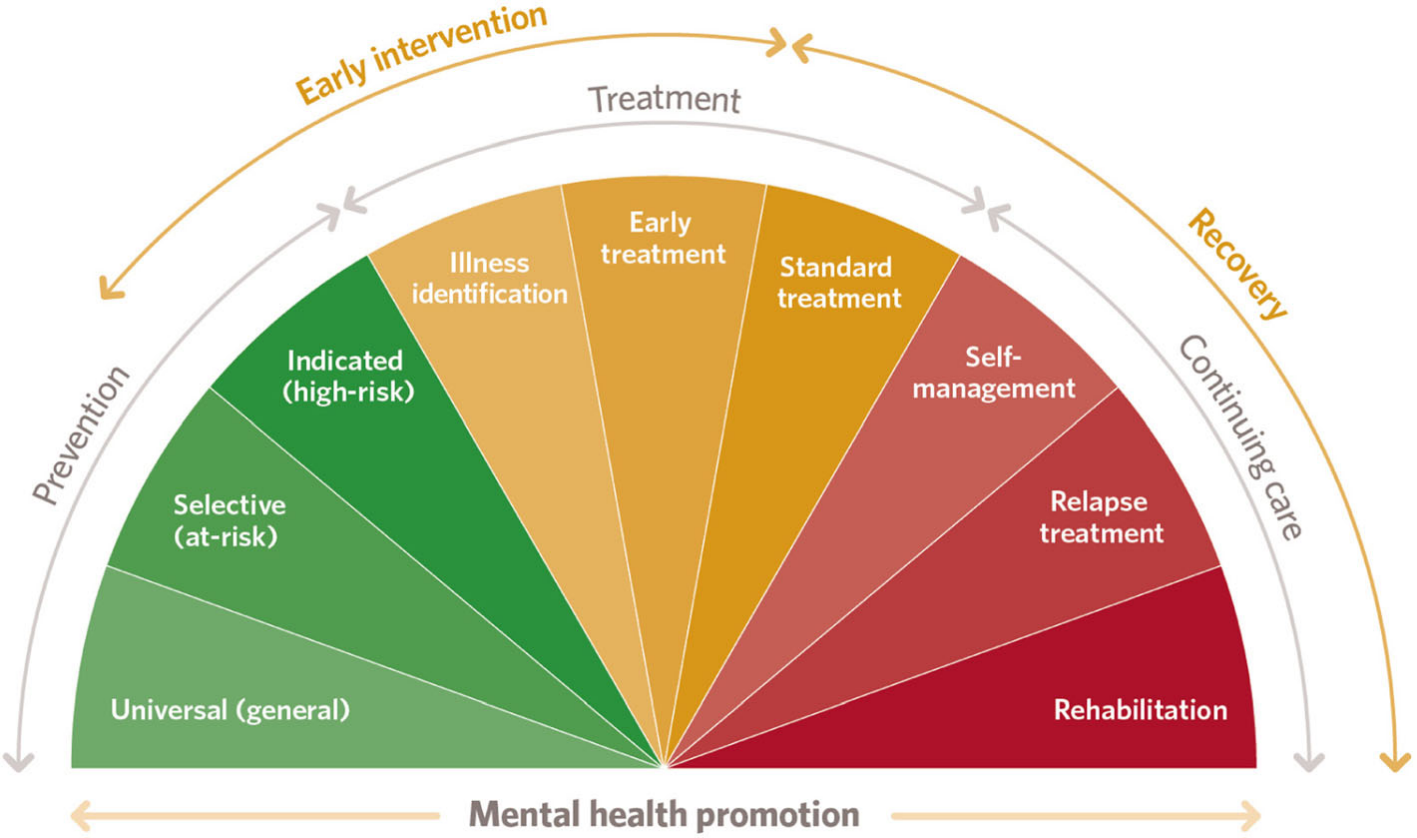
The mental health promotion spectrum

In addition to ecological factors, a comprehensive framework for mental health should encompass both prevention and early intervention, consistent with established models that are influential in public health and social policy (e.g. Haggerty and Mrazek’s mental health promotion spectrum [44]; see Fig. 2). An early intervention framework can optimise athlete mental wellbeing and respond rapidly to mental health symptoms and disorders as they emerge to best maintain the athlete’s overall function.

Within this framework, the prevention stages aim to reduce the risk of mental health symptoms developing or to minimise their potential impact and severity; the treatment and early intervention stages seek to identify and halt the progression of emerging mental health difficulties; and the continuing care stages help an individual to recover and prevent relapse, typically through ongoing clinical care with a mental health professional [44].

**Fig 3.**
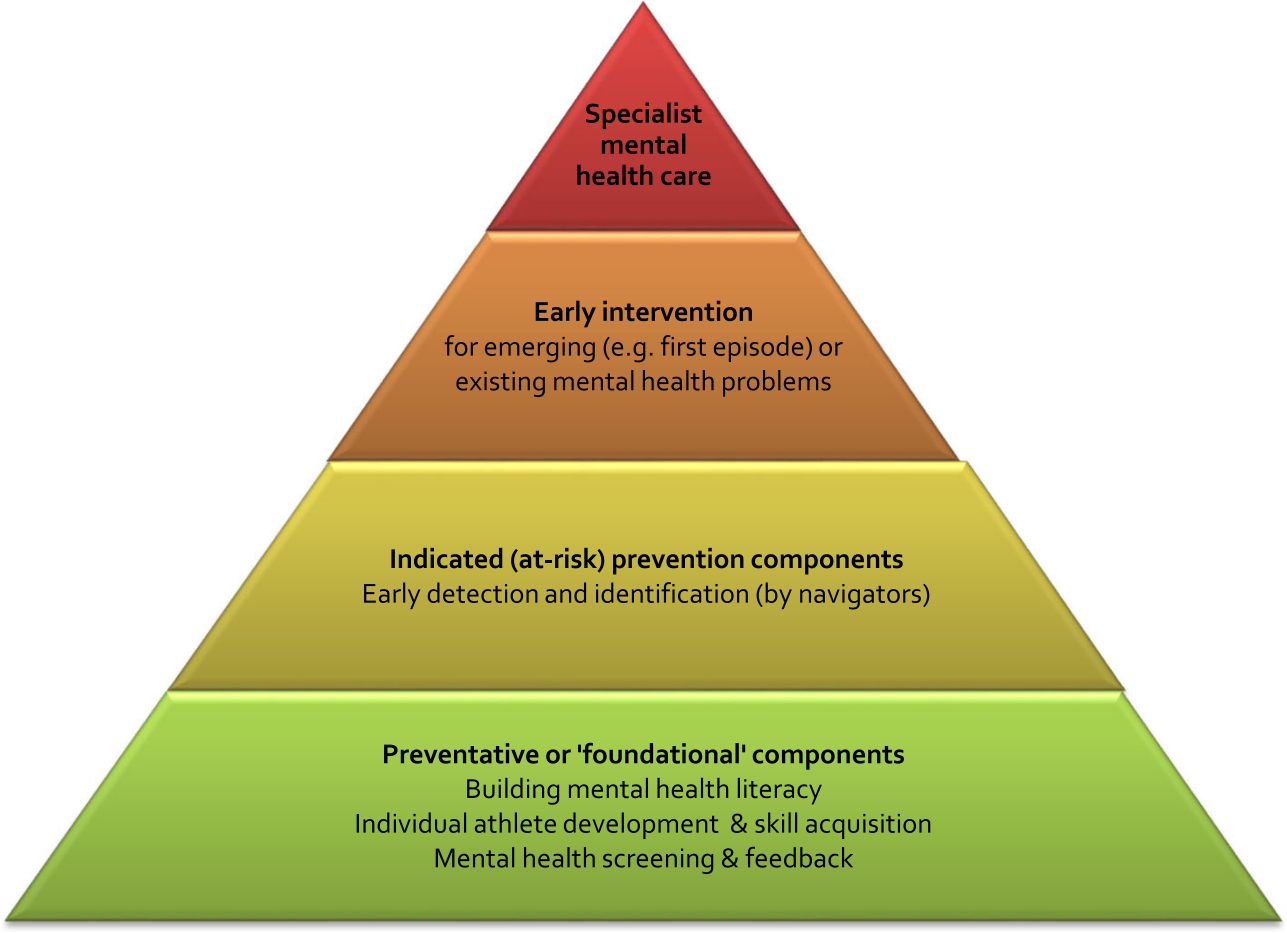
Elite athlete mental health and wellbeing framework

Based on the extant literature regarding risk factors for mental ill-health in elite athletes, along with existing sporting guidelines or statements regarding athlete wellbeing, and our experience developing and implementing early intervention services and system reform for young people’s mental health [45–47], we propose the following framework to respond to the mental health of elite athletes (see Fig. 3).

### Preventative or ‘Foundational’ Components

Core foundational components should include (i) mental health literacy to improve understanding, reduce stigma and promote early help-seeking; (ii) a focus on athlete development (both career and personal development goals) and skill acquisition to help attain these goals; and (iii) mental health screening of, and feedback to, athletes. The purpose of these foundational components is to enhance awareness of the importance of athlete wellbeing across the elite sport ‘ecology’. This in turn addresses workplace duty of care and occupational health and safety responsibilities towards athletes’ overall wellbeing in the context of sport-related stressors.

#### Mental Health Literacy

Mental health literacy programs should be provided to athletes, coaches and high-performance support staff to help to create a culture that values enhancing the mental health and wellbeing of all stakeholders. Programs should also be offered to the athlete’s family or friends to build their capacity to identify symptoms and encourage help-seeking, particularly as these are the individuals from whom athletes will initially seek help and support [48, 49]. Engaging an array of individuals, including organisational staff, in these programs broadens the reach of mental health literacy within an athlete’s (or sport’s) ecology (see Fig. 1). Gulliver and colleagues effectively trialled the delivery of a mental health literacy program to elite athletes via team-based workshops facilitated by mental health professionals [26]. This delivery method is preferred given the opportunity for qualified facilitators to discuss and explore athlete questions or concerns (especially regarding confidentiality and the implications of help-seeking for selection) and to potentially problem-solve together. The content of such training should be customised to address the specific aspects of the sport (e.g. team-based versus individual sport) and developmental stages (e.g. junior versus retiring athletes). Basic program content should cover (i) athlete-specific and general risk factors that can increase susceptibility to mental ill-health; (ii) key signs or symptoms of impaired wellbeing; (iii) how and from whom to seek help, both within and outside the sport; and (iv) basic techniques for athletes to self-manage transient mood states or psychological distress, such as relaxation techniques, adaptive coping strategies, self-compassion and mindfulness.

#### Individually Focused Development Programs

Individually focused development programs can assist athletes to identify personal/vocational goals and acquire the skills necessary to achieve them. This is necessary to help develop a parallel non-athletic identity, the skills to manage life-sport balance and to prepare for the eventual end of competitive sport. The latter may be challenging in younger athletes who often lack the longer-term perspective or life experience to perceive the need for such planning. However, a focus on developing a non-athletic identity must occur at all phases of the sporting career and not be confined to the transition out of sport phase, since building such skills takes time (and athletes are prone to unplanned retirement due to injury). These activities are ideally facilitated by a skilled, well-trained ‘peer workforce’. These are individuals who have a lived experience of mental ill-health and sufficient training to share their knowledge to help support others in similar situations [50]. In the sporting context, a peer workforce could include former athletes or coaches who work with current athletes to discuss and normalise experiences of mental health symptoms or their risk factors. Former athletes can assist with athlete development programs and mobilise athletes to the importance of actively participating with such programs, based on their own experiences [39].

#### Mental Health Screening

Mental health screening should be included alongside routine physical health checks by medical staff as part of a comprehensive framework. Screening items should be sensitive to the elite context [50, 51] and should be designed to provide feedback to athletes to help promote improved self-awareness, such as their mental state and triggers for symptoms. Critical times to screen are following severe injury (including concussion) and during the transition into, and out of sport [1], and the lead-up to and post major competitions may also be periods of higher risk. It is important to note that there is currently a lack of widely validated athlete-specific screening tools, though one elite athlete sensitised screening measure—the Athlete Psychological Strain Questionnaire—has been validated in a large sample of male elite athletes reporting strong psychometric properties [52], and is under further validation with female and junior athletes. Research potential exists to not only develop further athlete-specific measures, but to determine who is best suited to conduct screening, and what credentials or training may be required to ensure safety and integrity in this process (e.g. that appropriate help or referral is provided to athletes who screen positive).

### Indicated (‘at-risk’) Prevention Programs

The second phase is indicated prevention programs for those considered or assessed as being ‘at-risk’ of impaired mental health and wellbeing. This phase aims to mitigate the likelihood of deterioration in mental health by detecting symptoms as early as possible and facilitating referral to appropriate health professionals. Key staff within the sports system can be assisted to develop skills in early symptom identification and to promote professional help-seeking. This includes coaches, athletic trainers and teammates (where appropriate) who are in a position to notice ‘micro’ changes in an athlete over days or weeks, and sports medicine staff, such as physiotherapists who may detect other non-observable signs, such as changes in energy or body tension. We term these individuals ‘navigators’ in the mental health framework, as they have a crucial role in observing the athlete’s behaviour or mental state and being able to link them to professional care. These navigators can be provided with additional training (adjunctive to mental health literacy) to better recognise and interpret the athlete’s behaviour in relation to their overall wellbeing, understand athlete privacy concerns that inhibit the disclosure of mental health symptoms and build self-efficacy to be able to raise their concerns safely with the affected athlete or medical/mental health staff.

Sport administrators should also consider developing guides on ‘what to do if concerned about an athlete’s mental wellbeing’ and make these available to all relevant staff. These should include information regarding appropriate referral sources, responses (e.g. prevention program vs. early intervention) and facilitators to engage athletes, such as support and encouragement [27, 28] and/or linking mental wellbeing with athletic performance [29, 30]. Protocols or guides for responding to mental health concerns become less stigmatised when wellbeing needs are already routinely promoted via foundational programs.

### Early Intervention

Early intervention is necessary in instances where the performance and life demands placed on an athlete exceed their ability to cope (i.e. major career-threatening injury or significant life stress). Structured clinical interventions for mild to moderate mental ill-health are typically indicated at this phase and should ideally be provided ‘in-house’ by mental health clinicians, such as sports or clinical psychologists or psychiatrists, or medical staff where appropriate (e.g. pharmacotherapy). The use of in-house professionals helps to counter the low levels of service use associated with referring athletes out to external service providers and the stigma that is associated with the athlete needing expert ‘outside help’ [53]. Where requisite in-house expertise does not exist, this can be managed by the use of qualified consultants, but ideally these professionals should be ‘embedded’ to some extent within the sporting environment to ensure that athletes and other staff understand ‘who they are and what their role is’, even if their presence is infrequent [54]. When referral out is necessary, or preferred by the athlete, ideally this should be to a mental health professional with appropriate sport sensitised training, knowledge and experience assisting elite athletes.

Early interventions need not always be face-to-face, but can be augmented by telephone or web-enabled consultations, the latter particularly relevant given the frequency with which elite athletes travel unaccompanied by the sporting entourage. All interventions, regardless of the mode of delivery, should use an individualised care approach that is based on assessment and conceptualisation of the individual athlete’s presenting problem(s). The intervention should target the psychological processes of the athlete that are impeding mental health [55] and take account of the specific familial, sporting and organisational issues that may be impacting on the athlete’s wellbeing.

An example of an early intervention model of care is the Australian Institute of Sport (AIS) mental health referral network [56]. Athletes are assessed by an AIS mental health advisor, who can make a referral, if necessary, to a qualified mental health practitioner who has been credentialed to work within the network. This practitioner then works individually with the athlete to address their needs and ideally restore their mental health and functioning [57].

### Specialist Mental Health Care

Despite best efforts to prevent or intervene early, some athletes will nonetheless experience severe or complex psychopathology requiring specialist mental health care, particularly where there is a risk of harm to self or others. In some cases, this may include hospitalisation or specialist inpatient or day programs. The IOC Expert Consensus Statement provides a summary of recommended clinical interventions for a range of mental disorders, including bipolar, psychotic, eating and depressive disorders, and suicidality [1]. Developing and implementing a mental health emergency plan may also be required, particularly in cases where an athlete presents with an acute disturbance in their mental state, for instance agitation/paranoia, or suicidal ideation [58]. The IOC Expert Consensus Statement recommends that structured plans should acknowledge and define what constitutes a mental health emergency, identify which personnel (or local emergency services) are contacted and when, and consider relevant mental health legislation [1].

There is also arguably a need for ‘return to sport or training’ guidance for athletes who have been unable to compete or train for their sport due to mental illness, akin to guidelines for managing concussion [59]. Such guidance could potentially provide a graduated, step-by-step protocol that prepares not only the athlete for a successful return to sport, but also the microsystem that supports them.

## Conclusions

We have proposed a comprehensive framework for elite athlete mental health. More research is needed to bolster the efficacy of the approaches discussed here in the elite sports context, as well as other factors that are under-researched in the literature, such as gender-specific considerations in mental health [60] and considerations for para-athletes [23]. We are mindful that coaches and other high-performance staff are vulnerable to mental health problems [61] and the needs of these individuals need to be incorporated into a more inclusive model of care. Further, we recognise the scope of this framework does not cover the needs of non-elite athletes. Elements of this framework may be tailored in the future to be applicable and contextualised for non-elite environments where there may be limited resources, less professional staffing and greater limitations in athlete schedules.

Despite the exponential increase in research interest related to athlete mental wellbeing, major service delivery and treatment gaps remain. Evaluating the efficacy of mental health prevention and intervention programs via controlled trials or other high-quality designs is urgently needed. Program evaluation should ideally adopt an ecological systems approach to account for competition-related, individual-vulnerability and organisational factors on mental health outcomes, for example by seeking to measure system-level variables (e.g. the degree of perceived psychological safety within the sporting organisation [62, 63]) and individual athlete-level variables (e.g. coping skills, relationship with coach, injury history). As initiatives are evaluated and enhanced or adapted, developers should consult with elite sport organisations and individuals to ensure the relevance and sport sensitivity of their programs. Increased resources and research funding to support the evaluation and implementation of athlete mental health programs is needed, such as currently exists for managing athletes’ *physical* health (e.g. musculoskeletal injuries, concussion).

Finally, we are acutely aware that a framework such as that articulated here requires substantial investment and that such funding is scant even in high income settings. The foundational and at-risk components lend themselves, we believe, to be adaptable to low resource settings, given the emphasis on athlete self-management and a trained peer workforce. Adaptations to providing early intervention in low resource settings will be needed, and innovations in general mental health can act as a blueprint [64]. Regardless of settings or resources, investment in a comprehensive response to athlete mental health needs attention if it is to ever gain parity with physical health.

## Data Availability

Not applicable.

## Abbreviations

IOC: International Olympic Committee
AIS: Australian Institute of Sport
